# Endogenous Endophthalmitis from Urinary Tract Infection Caused by Group B Streptococcus: A Case Report

**DOI:** 10.3390/medicina60050700

**Published:** 2024-04-25

**Authors:** Heejeong You, Joonhyung Kim

**Affiliations:** Department of Ophthalmology, CHA Bundang Medical Center, CHA University School of Medicine, Seongnam 13496, Republic of Korea

**Keywords:** endogenous endophthalmitis, urinary tract infection, group B streptococcus

## Abstract

We present a case of endogenous endophthalmitis with urinary tract infection (UTI) caused by group B Streptococcus (GBS). An 86-year-old female initially presented with ocular pain and sudden visual disturbance of the left eye. The patient did not complain of other symptoms and had no history of recent ocular surgery or trauma. Endogenous endophthalmitis was clinically diagnosed based on ophthalmic examination, history, and lab results showing systemic infection. A few days later, GBS was identified in her aqueous humor, blood, and urine cultures. Intravitreal ceftazidime and vancomycin injections, as well as fortified ceftazidime and vancomycin eye drops, were used immediately after clinical diagnosis. However, the symptoms worsened despite repeated intravitreal injections, so evisceration was performed. Endogenous endophthalmitis caused by GBS is very virulent and may present without evident systemic symptoms. The early recognition of the disease and systemic work up, followed by prompt treatment, is necessary.

## 1. Introduction

Endogenous endophthalmitis is an intraocular infection caused by the hematogenous dissemination of infectious microorganisms from a distant infection site. Due to its systemic pathophysiology, endogenous endophthalmitis is related to immunosuppressive states, including chronic diseases such as diabetes mellitus, intravenous drug abuse, and indwelling catheters [1]. The causative organisms of endogenous endophthalmitis vary by geographical region. *Staphylococcus aureus* and *Streptococcus pneumoniae* are prevalent in Western countries, and *Klebsiella pneumoniae* is the most common cause in Eastern countries [2].

*Streptococcus agalactiae*, also known as ß-hemolytic group B Streptococcus (GBS), is a rare cause of endogenous endophthalmitis, particularly in East Asia [3]. Previous case reports and literature reviews revealed poor visual prognosis, especially with poor initial visual acuity [4,5]. Endocarditis was the most common infection focus, followed by arthritis, cellulitis, and urinary tract infection (UTI) [5].

We report a case of an 86-year-old woman who initially presented with ophthalmic symptoms as the first subjective symptom of endogenous endophthalmitis from UTI caused by GBS.

## 2. Case Report

**Figure 1:**
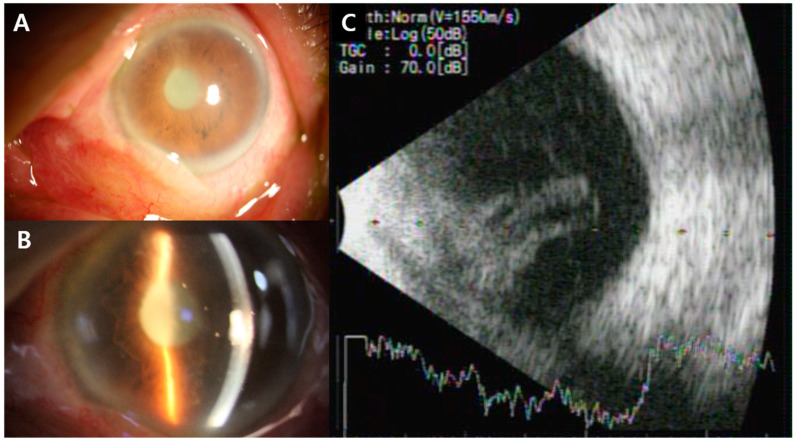
(**A**) Anterior segment photo of the patient. There were conjunctival injections and corneal edema. (**B**) Anterior segment photo of the patient. There were grade 4+ anterior chamber cells with fibrinous membranes completely blocking the pupil, and some roundish inflammatory materials were visible. (**C**) B-scan of the patient revealed vitreous opacity.

An 86-year-old female was referred from the emergency department for ocular pain and visual disturbance of the left eye from the day before. Her vision was 20/30 in the right eye and no light perception in the left eye. Intraocular pressure, measured using a Goldmann applanation tonometer, was 10 mmHg in the right eye and 34 mmHg in the left eye. A slit lamp examination of the left eye revealed lid swelling, conjunctival injection, corneal edema, and anterior chamber cell 4+ with fibrinous membranes completely blocking the pupil (Figure 1A,B). Fundus examination was impossible due to fibrinous membranes, and vitreous opacity was observed in the B-scan ultrasonogram (Figure 1C).

The patient denied any previous ocular trauma, infection, or surgery, except for bilateral cataract surgeries carried out 20 years ago. She was regularly followed up for hypertension, diabetes, and angina. Although the patient did not report febrile sensations, her body temperature was 37.6 °C. Her white blood cell count was 15.52 × 10^9^/L, and the erythrocyte sediment rate and C-reactive protein levels were markedly increased at 114 mm/h and 14.89 mg/dL, respectively. Electrolytes, liver enzymes, and kidney function results were within the normal range, except for mild hyponatremia. The urinalysis showed a positive WBC, suggesting the possibility of systemic infection starting from urinary tract infection. No other acute infectious conditions were noted in the chest and abdominopelvic CT.

Based on the ophthalmic examination, patient history, and lab results, she was diagnosed with endogenous endophthalmitis. Immediate intravitreal vancomycin (1 mg/0.1 mL) and ceftazidime (2.25 mg/0.1 mL) injections were carried out, and fortified vancomycin and ceftazidime eye drops were also administered every hour. Intravenous ceftriaxone and vancomycin were given to control the systemic infection. Blood culture, urine culture, and anterior chamber tap culture all revealed *Streptococcus agalactiae*, a β-hemolytic group B Streptococcus bacterium. The isolated pathogen was susceptible to both ceftriaxone and vancomycin, so intravenous vancomycin was stopped and only ceftriaxone was administered. No endocarditis was noted in transesophageal echocardiography.

**Figure 2:**
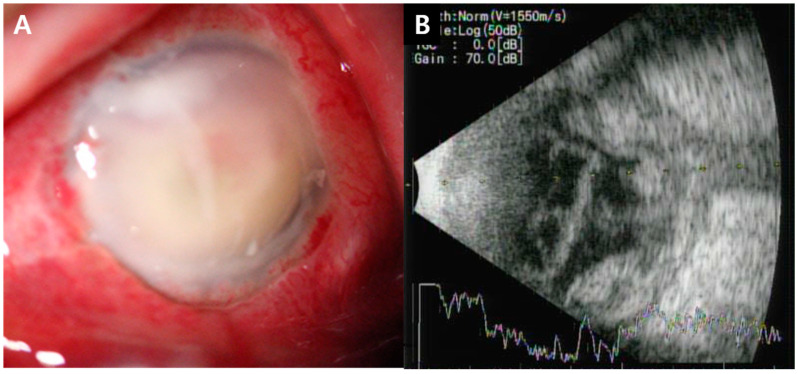
(**A**) Conjunctival chemosis and injection, corneal edema, and anterior chamber empyema. (**B**) B-scan showing much increased vitreous opacity with retinal detachment.

**Figure 3:**
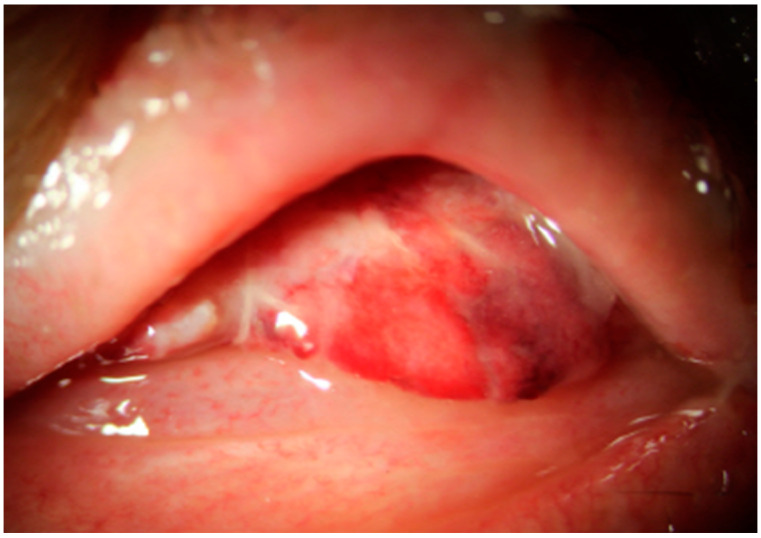
Anterior segment photo of the patient after evisceration.

Intravitreal vancomycin and ceftazidime injections were carried out five times every two or three days. Despite repeated intravitreal antibiotics injections, her visual acuity still had no light perception, and anterior chamber empyema and vitreous opacity continued to increase; moreover, retinal detachment was observed in the follow-up B-scan (Figure 2). Therefore, evisceration with hydroxyapatite was performed (Figure 3), and eyelid swelling and eyeball pain were resolved.

## 3. Discussion

Regarding all types of endophthalmitis, endogenous endophthalmitis itself is a risk factor for poor outcomes, needing evisceration or enucleation [6]. Previous studies reported good initial visual acuity, pars plana vitrectomy, and intravitreal injection within the first 24 h of diagnosis as better visual prognostic factors for endogenous endophthalmitis [7]. While early vitrectomy has recently been widely accepted for exogenous endophthalmitis, there is no definite consensus about vitrectomy for endogenous endophthalmitis [8,9,10]. Our patient already had no light perception at the initial presentation, and since her general condition was very poor, vitrectomy was not our priority consideration. Although our patient received intravitreal antibiotic injections immediately at her initial presentation and repeatedly thereafter, the course of the disease was very virulent, and evisceration was inevitable.

Although our patient’s infection focus was UTI, she did not complain of any symptoms. A previous study reported that 19% of endogenous endophthalmitis patients with *Klebsiella pneumoniae* liver abscesses initially presented to an ophthalmology clinic [11,12]. Ophthalmologists should note that ocular manifestations caused by endogenous endophthalmitis can be the first symptoms of systemic infection. Therefore, prompt evaluation for sepsis and infection focus, such as pyogenic liver abscess, endocarditis, meningitis, UTI, and osteomyelitis should be initiated when there is no suspected history of exogenous endophthalmitis [13].

UTI is reported to be 5–17.5% of the primary source of infection in endogenous endophthalmitis, especially in immunocompromised patients [13,14,15,16,17,18]. *Klebsiella pneumoniae*, *E. coli*, and *Candida* are well-known microorganisms causing UTI and complicated endogenous endophthalmitis [7,13]. Ren et al. [19] reported a recent literature review of endogenous endophthalmitis caused by UTI and revealed that almost half of the patients had diabetes mellitus. Our patient also had diabetes mellitus.

GBS is not a typical causative microorganism for UTI and associated endogenous endophthalmitis, but recently, invasive infection in the elderly population, especially in patients with underlying chronic illnesses, such as diabetes mellitus, malignancy, and acquired immunodeficiency, is increasing [4,5]. Endogenous endophthalmitis caused by GBS is reported to have a poor prognosis. Yoshida et al. [5] reported that 60% of patients experienced complete vision loss, such as no light perception, phthisis bulbi, enucleation or evisceration, or death, and 7 out of 15 cases who underwent vitrectomy still resulted in complete vision loss.

## 4. Conclusions

This case highlights vigorously aggravating endogenous endophthalmitis from UTI caused by GBS despite prompt and precise diagnosis and intravitreal antibiotic injections. Even in the absence of evident systemic symptoms, an evaluation for systemic infection is important when endogenous endophthalmitis is suspected. Also, considering our experience, we recommend considering early vitrectomy for rapidly progressing endogenous endophthalmitis with limited response to intravitreal injections.

## Data Availability

The data presented in this study are available on request from the corresponding author. The data are not publicly available due to privacy.
